# Association between physical activity and conversion from mild cognitive impairment to dementia

**DOI:** 10.1186/s13195-020-00707-1

**Published:** 2020-11-11

**Authors:** Yeo Jin Kim, Kyung-Do Han, Min Seok Baek, Hanna Cho, Eun Joo Lee, Chul Hyoung Lyoo

**Affiliations:** 1 Department of Neurology, Chuncheon Sacred Heart Hospital, Hallym University College of Medicine, Chuncheon, Republic of Korea; 2 Department of Biostatics, College of Medicine, The Catholic University of Korea, Seoul, Republic of Korea; 3 Department of Neurology, Gangnam Severance Hospital, Yonsei University College of Medicine, 211, Eonju-ro, Gangnam-gu, Seoul, 06273 Republic of Korea; 4 Big Data Steering Department, National Health Insurance Service, Wonju, Republic of Korea

**Keywords:** Mild cognitive impairment, Physical activity, Continuance, Regularity

## Abstract

**Background:**

Physical activity has been suggested to prevent the conversion of mild cognitive impairment (MCI) to dementia in patients. We investigated the association between the continuance and regularity of physical activity and the risk of developing dementia in patients with MCI.

**Methods:**

We analyzed 6-year followed up data for 247,149 individuals in the National Health Insurance Service (NHIS) cohort of Korea who were enrolled between January 1, 2009, and December 31, 2015. The patients were divided into four groups: those who did not engage in physical activity consistently (Never-PA group), those who initiated physical activity (Initiation-PA group), those who ceased physical activity (Withdrawal-PA group), and those who performed physical activity consistently (Maintenance-PA group). We also divided the patients into two groups: those who engaged in physical activity irregularly (Irregular-PA) and those who undertook physical activity regularly (Regular-PA).

**Results:**

When the risk for the Never-PA group was set as the benchmark (ref = 1), the Maintenance-PA group had the lowest incidence of dementia of the Alzheimer type (DAT) compared to the other groups (HR = 0.82, 95% CI 0.79–0.86). The DAT risk of the Initiation-PA group (HR = 0.89, 95% CI 0.85–0.93) was lower than the Never-PA group. In addition, compared to the Irregular-PA group, the Regular-PA group had a 15% reduced risk for developing DAT.

**Conclusions:**

Although no causal inference could be made, continued regular physical activity in patients with MCI is associated with a protective effect against developing DAT. Moreover, ceasing physical activity could halt this protective effect.

**Supplementary information:**

**Supplementary information** accompanies this paper at 10.1186/s13195-020-00707-1.

## Background

Mild cognitive impairment (MCI) is associated with a high risk of progression to dementia [1–5]. There are currently no approved disease-modifying treatments, and lifestyle modifications have become an important strategy to prevent its progression. Physical activity is considered to be the most important interventional strategy for the prevention of dementia, with previous studies showing that physical activity reduces the likelihood of disease progression in early dementia [6].

The types, intensity, duration, and frequency of physical activity have been shown to impact the protective effect of physical activity [7]. Leisure-time physical activity is associated with a reduced risk of dementia, while work-related physical activity has not shown an association with a protective effect [8]. High-intensity physical activity was found to be more effective than low-intensity physical activity [9], while the duration and frequency of physical activity is also an important factor that can reduce the risk of cognitive decline. In a recent meta-analysis, physical activity of more than 16 weeks was associated with a greater improvement in cognition [10]. In addition, the incidence rate of dementia was lower in subjects who engaged in physical activity more than twice per week [11, 12]. However, the association between withdrawal or initiation of physical activity and prevention against cognitive decline remains poorly understood. Similarly, the importance of the regularity of physical activity needs further investigation. We thus sought to investigate the effect of physical activity continuance and regularity on the rate of conversion from MCI to dementia of the Alzheimer type (DAT) using longitudinal data from the National Health Insurance Service (NHIS) covering Korean patients that were followed up for 6 years.

## Methods

### Data source and study population

The present study was conducted using data from the national health insurance claims database established by the National Health Insurance Service (NHIS) of Korea [13]. The NHIS registration is mandatory for all Koreans, except for those with low incomes. People with low incomes are enrolled in the Medical Aid program; however, since 2006, these patient data have also been incorporated into the NHIS database. Therefore, the information obtained from the NHIS database represents health information for almost all patients in Korea [14].

**Fig. 1:**
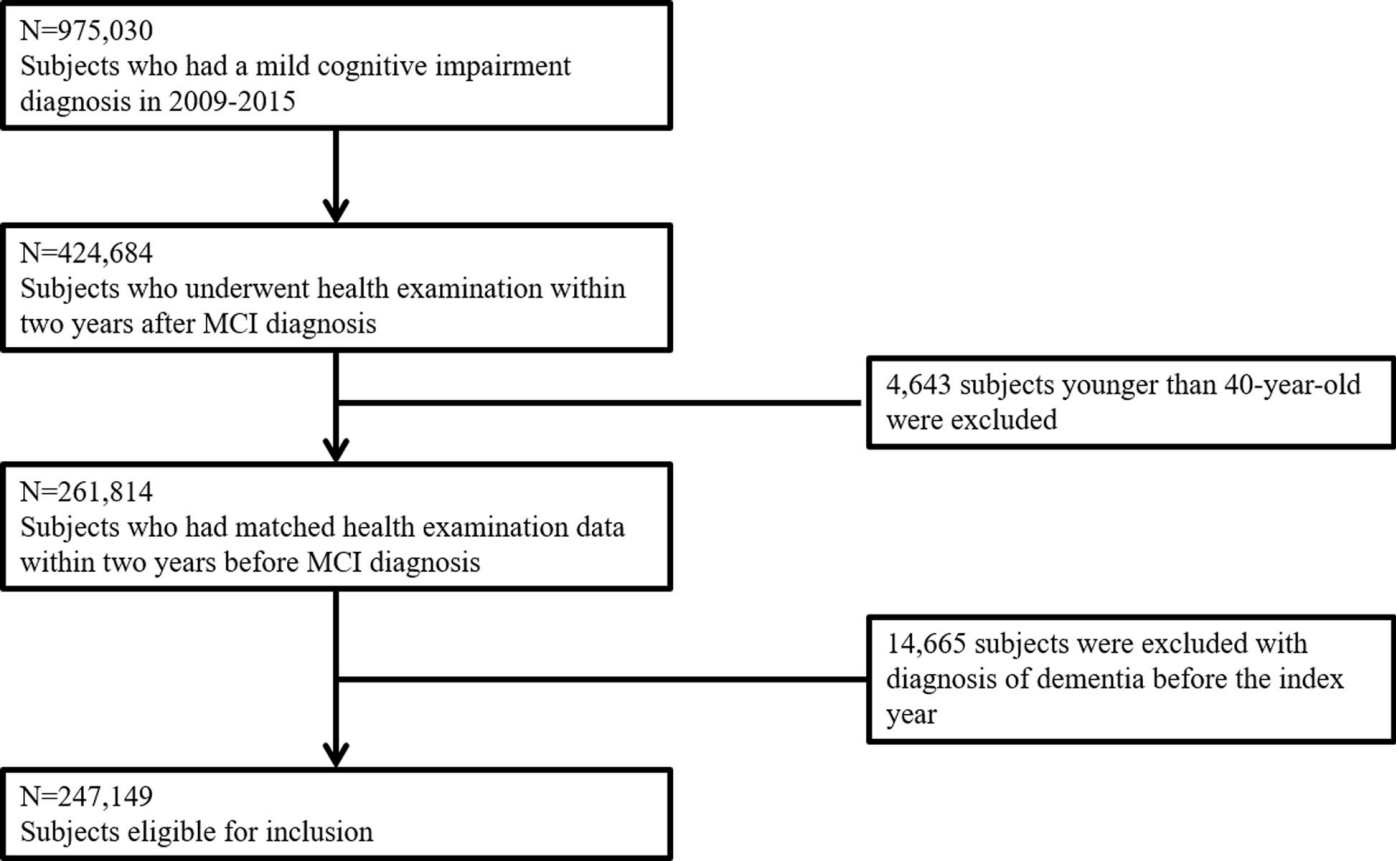
Flow chart of the study population

The NHIS database includes demographic information, medical use, disease information, lifestyle habits, and basic laboratory data [15]. We defined diagnoses using the International Classification of Disease, Tenth Revision, Clinical Modification (ICD-10-CM) codes. The general health examination is administered to all citizens over 20 years every 2 years. Data from all patients with an MCI diagnosis from January 1, 2009, to December 31, 2015, were included (*n* = 975,030). Of these MCI patients, we selected only those who had undertaken a health examination within 2 years after their MCI diagnosis (*n* = 424,684). We excluded patients aged < 40 years (*n* = 4643) and only selected those with matched examination data within 2 years before their MCI diagnosis (*n* = 261,814). Patients with dementia diagnosed before the index date were excluded (*n* = 14,665). The index date of this study was second health examination date. In the end, 247,149 individuals were included in this study (Fig. 1). The period from 1st health examination to MCI diagnosis was 12.0 months and the period from MCI diagnosis to 2nd health examination was 10.8 months (Supplementary Table 1).

### Standard protocol approvals, registrations, and patient consent

We obtained written informed consent from each patient. This study was approved by the Institutional Review Board of Gangnam Severance Hospital, and all methods were performed in accordance with the approved guidelines and regulations.

### Definition of MCI and DAT

MCI was diagnosed using the ICD-10-CM codes and includes F067. The codes for DAT were F00 and G30.

### Physical activity measurements

Data on physical activity were collected via self-report questionnaires. The extent of physical activity was assessed using the Korean version of the international physical activity questionnaire (K-IPAQ) short form, focusing on physical activity performed in the last 7 days. The 7 items of the IPAQ identify the total minutes over the last 7 days spent on moderate- and vigorous-intensity physical activity. Participants were asked how many days per week they spent for each activity over 10 min and how many minutes per activity they spent per day over the last 7 days. Vigorous activities included carrying heavy objects, running, aerobics, and fast biking. Moderate activities included carrying light items, bike riding at normal speed, and doubles tennis.

### Classification of MCI patients

**Fig. 2:**
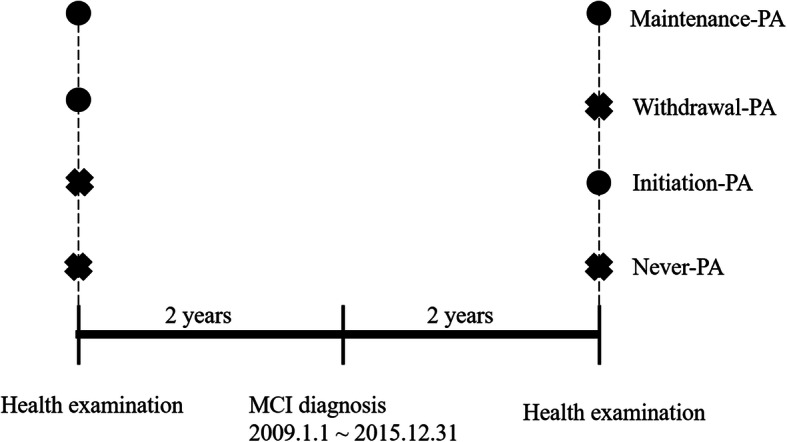
Classification of the four groups of MCI patients. The circle indicated that the patient had performed moderate to vigorous physical activity in the last 7 days. The cross indicated that the patient had not performed physical activity in the last 7 days

We classified patients with MCI into four groups (Fig. 2). The “Never-PA” group included those patients who did not engage in physical activity before or after MCI diagnosis. We defined the physical activity to be vigorous or moderate physical activity over 10 min more than 1 days per week. The “Initiation-PA” group included patients who did not engage in physical activity before the diagnosis of MCI but initiated physical activity after the diagnosis of MCI. The “Withdrawal-PA” group included patients who engaged in physical activity before the diagnosis of MCI but stopped physical activity after the diagnosis of MCI. The “Maintenance-PA” group included patients who engaged in physical activity before and after MCI diagnosis. We also classified patients with MCI into two groups according to the regularity of physical activity. The “Regular-PA” group included patients who performed physical activity regularly, while the “Irregular-PA” group included patients who engaged in physical activity irregularly. We defined regular physical activity to be vigorous physical activity more than 3 days per week or moderate physical activity more than 5 days per week.

### Statistical analysis

The data from the NHIS database are presented as mean values ± standard deviation (SD) for continuous variables and percentages for categorical variables. Differences between groups were confirmed using analysis of variance (ANOVA) for continuous variables and chi-square test for categorical variables. Cox proportional hazard models were used to assess the risk of dementia. Dementia risk was expressed as the hazard ratio (HR) with 95% confidence interval (95% CI). Incidence rates were calculated per 1000 person-years. *P* values were two-tailed, and statistical significance was considered at *P* < 0.05. All statistical analyses were performed using SAS V.9.3.

## Results

### Demographics and baseline characteristics

Detailed demographic and clinical characteristics of the participants are presented in Table 1. The mean age of the patients in the Never-PA group was highest, while the mean age of the patients in the Maintenance-PA group was lowest. The Never-PA group had the highest proportion of females. In addition, the Never-PA group had the highest proportion of patients with hypertension or diabetes mellitus. Compared to the Irregular-PA group, the Regular-PA group is younger and had the higher proportion of females. The Irregular-PA group had the higher proportion of patients with hypertension (Supplementary Table 2).

**Table 1:** Baseline characteristics of the study population

	Never-PA (*n* = 99,873)	Initiation-PA (*n* = 45,598)	Withdrawal-PA (*n* = 45,014)	Maintenance-PA (*n* = 56,664)	*P* value
Age, year	68.7 ± 9.3	66.4 ± 9.2	67.2 ± 9.1	64.6 ± 9.1	< 0.0001
Female, *n* (%)	72,836 (72.9)	30,862 (67.7)	30,131 (66.9)	32,204 (56.8)	< 0.0001
Hypertension, *n* (%)	58,178 (58.3)	24,777 (54.3)	24,889 (55.3)	28,962 (51.1)	< 0.0001
Diabetes mellitus, *n* (%)	22,448 (22.5)	9542 (20.9)	10,062 (22.4)	11,171 (19.7)	< 0.0001
Dyslipidemia, *n* (%)	45,981 (46.0)	20,976 (46.0)	20,893 (46.4)	25,596 (45.2)	0.001
Obesity*, *n* (%)	37,096 (37.1)	16,617 (36.4)	16,407 (36.5)	20,160 (35.6)	< 0.0001
Body mass index, kg/m^2^	24.16 ± 3.25	24.15 ± 3.05	24.15 ± 3.10	24.13 ± 2.88	0.257
Smoking, *n* (%)					< 0.0001
Non-smoker	82,345 (82.5)	36,071 (79.1)	36,017 (80.0)	40,576 (71.6)	
Ex-smoker	9812 (9.8)	6093 (13.4)	5665 (12.6)	11,689 (20.6)	
Current smoker	7716 (7.7)	3434 (7.5)	3332 (7.4)	4399 (7.8)	
Drinking, *n* (%)					< 0.0001
Non-drinker	84,755 (84.9)	35,743 (78.4)	36,858 (81.9)	39,453 (69.6)	
Mild to moderate drinker	12,598 (12.6)	8593 (18.9)	6953 (15.5)	15,241 (26.9)	
Heavy drinker	2520 (2.5)	1262 (2.8)	1203 (2.7)	1970 (3.5)	
Time to conversion from MCI to dementia (months)	31.3 ± 16.6	31.6 ± 16.7	31.6 ± 16.8	31.0 ± 16.4	< 0.0001

*PA* physical activity

*Body mass index ≥ 25 kg/m^2^

### Risk of dementia according to continuity of physical activity after diagnosis of mild cognitive impairment

Of the 247,149 patients analyzed, 23,015 patients converted to dementia. Of these 23,015, 17,733 (77%) were diagnosed with DAT.

Table 2 shows the risk of DAT according to the continuity of physical activity. The Maintenance-PA group showed the lowest risk of dementia. Compared to the Never-PA group, the risk of DAT was reduced in the Initiation-PA group and Maintenance-PA group (for the Initiation-PA group: HR 0.89, 95% CI 0.85–0.93; for the Maintenance-PA group: HR 0.82, 95% CI 0.79–0.86), while the risk of DAT was the same between the Never-PA group and Withdrawal-PA group (for Withdrawal-PA group: HR 1.00, 95% CI 0.96–1.04). There were no interactions between the comorbidity and the continuity of physical activity (Supplementary Table 3). The association between initiating physical activity and dementia risk in females was greater than in males, while the association between continuance of physical activity and dementia risk in males was greater (in females for the Initiation-PA group: HR 0.88, 95% CI 0.84–0.92; for Withdrawal-PA group: HR 0.99, 95% CI 0.94–1.03; for Maintenance-PA group: HR 0.82 95% CI 0.78–0.86; in males for the Initiation-PA group: HR 0.92, 95% CI 0.86–0.97; for Withdrawal-PA group: HR 0.98, 95% CI 0.92–1.04; for Maintenance-PA group: HR 0.77 95% CI 0.73–0.82). However, these gender-specific differences were not statistically significant.

**Table 2:** Risk of dementia according to continuity of physical activity

	Never-PA (*n* = 99,873)	Initiation-PA (*n* = 45,598)	Withdrawal-PA (*n* = 45,014)	Maintenance-PA (*n* = 56,664)
DAT cases, *n* (%)	8658 (8.7%)	2888 (6.3%)	3445 (7.7%)	2742 (4.8%)
DAT incidence (per 1000 person-years)	33.25	24.11	29.13	18.77
Unadjusted HR (95% CI)				
DAT	1 (ref)	0.73 (0.70–0.76)	0.88 (0.84–0.91)	0.56 (0.54–0.59)
Adjusted HR* (95% CI)				
DAT	1 (ref)	0.89 (0.85–0.93)	1.00 (0.96–1.04)	0.82 (0.79–0.86)

*HR* hazard ratio, *CI* confidence interval, *DAT* dementia of the Alzheimer type, *PA* physical activity

*Adjusted for age, gender, hypertension, diabetes mellitus, dyslipidemia, body mass index, history of smoking, and history of alcohol intake

### Risk of dementia according to regularity of physical activity

Table 3 shows DAT risk according to the regularity of physical activity. When the risk for the Irregular-PA group was set as a reference (ref = 1), compared to the Irregular-PA group, the Regular-PA group had a 15% reduced risk of Alzheimer’s disease after adjusting for age, gender, and vascular risk factors (HR 0.85, 95% CI 0.81–0.88).

**Table 3:** Risk of dementia according to regularity of physical activity

	Irregular-PA	Regular-PA*
Unadjusted HR (95% CI)		
Dementia of the Alzheimer type	1 (ref)	0.70 (0.68–0.73)
Adjusted HR (95% CI)^†^		
Dementia of the Alzheimer type	1 (ref)	0.85 (0.81–0.88)

*HR* hazard ratio, *CI* confidence interval, *PA* physical activity

*Vigorous-intensity physical activity ≥ 3 days per week or moderate-intensity physical activity ≥ 5 days per week

^†^Adjusted for age, gender, hypertension, diabetes mellitus, dyslipidemia, body mass index, history of smoking, and history of alcohol intake

## Discussion

Our nationwide population-based longitudinal cohort study analysis shows that continued physical activity in patients with MCI is associated with a lower risk of DAT. It appears that the decision to start physical activity leads to a lower risk of DAT, while ceasing physical activity may cause the risk of DAT to increase again. In addition, a higher frequency of physical activity appears to prevent conversion from MCI to DAT (moderate-intensity physical activity more than 5 days per week or vigorous-intensity physical activity more than 3 days per week).

We observed that the Maintenance-PA group had 18% fewer dementia conversions than the Never-PA group, while the Initiation-PA group had 11% less dementia conversion than the Never-PA group. We interpret this to indicate that continuing physical activity occurring at both time points was more effective than initiating a new physical activity between the two time points. Evidence suggests that the longer the duration of physical activity, the greater the effect of physical activity on cognitive function [16]. The findings for the Initiation-PA group may therefore reflect the shorter duration of physical activity compared to the Maintenance-PA group.

There are at least two major mechanisms by which continuous physical activity may prevent the conversion from mild cognitive impairment to dementia. Physical activity increases the expression of neurotrophic factors such as brain-derived neurotrophic factor (BDNF), insulin-like growth factor 1 (IGF-1), and vascular endothelial growth factor (VFGF). BDNF is important for maintaining neuronal development and for exercise-related improvements in cognitive function. IGF-1 and VFGF play important roles in neurogenesis and angiogenesis and influence the induction of hippocampal BDNF. Animal and clinical studies have demonstrated that physical activity can increase neurotrophic factors such as BDNF and IGF-1. After physical activity, the release of BDNF from the brain is enhanced [17, 18], with 6 months of resistance exercise sufficient to increase serum levels of IGF-1 in older adults [19].

Physical activity also increases cerebral blood flow (CBF). After 12 weeks of physical activity, CBF has been shown to increase in the anterior cingulate cortex [20] and hippocampal CBF increased in elderly patients with subjective memory complaints after 16 weeks of physical activity [21]. CBF is thought to maintain cerebral perfusion to help maintain brain volume. Previous studies have shown that physical activity is associated with increased regional gray and white matter volume including areas such as the hippocampus, and prefrontal and cingulate cortices [22, 23].

Ceasing regular physical activity appears to halt the positive effects on the body that the physical activity was eliciting. One small study found that elderly subjects who undertook endurance training for more than 15 years had reduced CBF in specific brain regions including the hippocampus after stopping physical activity [24]. Stopping physical activity also reduces cardiorespiratory fitness and muscle mass and increases glucose intolerance which might adversely affect cognitive function [25–27]. These adverse effects associated with stopping physical activity arise within 10–20 days. Our findings also showed that the dementia conversion rate was lower in the Initiation-PA group than the Withdrawal-PA group, so the duration of the positive effects of physical activity does not appear to be very long.

We also investigated the association between regularity of physical activity and dementia risk. The Regular-PA group showed a 15% reduction in dementia conversion compared to the Irregular-PA group. However, not all studies have shown that physical activity improves cognitive function, and the effects of exercise on MCI have shown inconsistencies. In a review of 14 randomized controlled trials, the majority had insufficient evidence of the benefits of physical activity on cognitive function in patients with MCI [28], whereas another review showed that aerobic exercise elicited beneficial effects on cognitive function in MCI patients [29]. A possible explanation for these discrepancies is that each study involved different conditions including the type, intensity, frequency, and duration of physical activity. There have also been three large multidomain trials, FINGER [30], MAPT [31], and PreDIVA [32], which sought to investigate whether lifestyle modifications prevent cognitive impairment. Among these studies, only the FINGER study showed positive results, which could be because it involved active physical training that was different to the PreDIVA and MAPT studies.

Effects of physical activity on cognitive function in demented patients were also unclear. A randomized control study reported that moderate- to high-intensity exercise training could not slow cognitive impairment in patients with mild to moderate dementia [33]. Many of studies included in a meta-analysis of randomized control trials showed beneficial effects of physical activity on cognitive function in patients with dementia. However, because of unclear methodology, it was difficult for these studies to conclude that the effect of physical activity on dementia patients was proved. On the other hand, several relatively large studies included in the meta-analysis reported no effects on global cognition [34].

We note several limitations to our study. First, the evaluation of physical activity was based on self-reporting by the participants and could be open to bias. Second, when we analyzed the association frequency of physical activity with dementia, we could not control for disease severity due to a lack of cognitive function information. However, since the subjects were diagnosed with mild cognitive impairment, the level of baseline cognition would presumably not be very different. Third, the participants’ physical activity characteristics, such as type, intensity, duration, and frequency, were free to change during the study period, but we did not take into account the effect of such changes. Moreover, we only measured the presence or absence of physical activity at two distinct time points. Fourth, the biomarker information could not be used for diagnosis. From the claim data used in this study, only clinical diagnosis based on ICD-10 could be obtained. Therefore, some of patients with dementia of the Alzheimer type in this study might be non-Alzheimer’s disease patients. Fifth, because of the data access policy, it was not possible to use data for people not included in the study, so it was impossible to perform additional analyses on people who were not included in the inclusion criteria, such as those who had health examination only once or who did not be diagnosed MCI. In addition, we did not investigate the relation between physical activity and incident MCI. Sixth, although this study had a longitudinal design, it was difficult to have causal inference. Since this was an observational study, possible confounder might cause the effect. Lastly, as the cohort included participants of Korean ethnicity, caution should be taken if generalizing these findings to other populations. However, our study involved a very large sample size of over 247,000 adults. In addition, we focused on the continuity and regularity of physical activity, representing more specific factors that can reduce the risk of DAT.

## Conclusion

Our study showed that continued physical activity in patients with MCI is associated with a lower risk of DAT. It appears that the decision to start physical activity leads to a lower risk of DAT, while ceasing physical activities may cause the risk of DAT to increase again. In addition, regular physical activity appears to prevent conversion from MCI to DAT. However, further research is needed into the duration of benefit from physical activity and the potential biological mechanisms involved. In addition, prospective studies are needed to clarify the result of this study.

## Supplementary information

**Additional file 1: Supplementary Tables.** **Supplementary Table 1.** Pre- and post-MCI diagnosis period in each group. **Supplementary Table 2.** Baseline characteristics of the study population between Irregular and Regular-PA. **Supplementary Table 3.** Interactions of comorbidities with continuity of physical activity.

## Data Availability

The original anonymized data used in this analysis was obtained from NHIS of Korea. The dataset from NHIS is not publicly available due to restricted access. However, any researcher requiring access to the data can obtain it directly through a license agreement, including the payment of appropriate license fees.

## Abbreviations

MCI: Mild cognitive impairment
DAT: Dementia of the Alzheimer type
NHIS: (Korean) National Health Insurance Service
ICD-10-CM: International Classification of Disease, Tenth Revision, Clinical Modification
K-IPAQ: Korean version of the international physical activity questionnaire
PA: Physical activity
SD: Standard deviation
ANOVA: Analysis of variance
HR: Hazard ratio
95% CI: 95% confidence interval
BDNF: Brain-derived neurotrophic factor
IGF-1: Insulin-like growth factor 1
VFGF: Vascular endothelial growth factor
CBF: Cerebral blood flow
FINGER: The Finnish Geriatric Intervention Study to Prevent Cognitive Impairment and Disability
MAPT: The Multidomain Alzheimer Preventive Trial
PreDIVA: The Prevention of Dementia by Intensive Vascular Care
