# Supplementary material for: Association between physical activity and conversion from mild cognitive impairment to dementia
Source: Alzheimers Res Ther. 2020 Nov 11;12:136. doi: 10.1186/s13195-020-00707-1 (PMC7661208; doi:10.1186/s13195-020-00707-1)
Supplement: Supplementary file 1 — Additional file 1: Supplementary Tables. Supplementary Table 1. Pre- and post-MCI diagnosis period in each group. Supplementary Table 2. Baseline characteristics of the study population between Irregular and Regular-PA. Supplementary Table 3. Interactions of comorbidities with continuity of physical activity. [file 13195_2020_707_MOESM1_ESM.doc]

Supplementary Table 1. Pre- and post-MCI diagnosis period in each group

|  | Pre-MCI diagnosis period | Post-MCI diagnosis period |
| --- | --- | --- |
| Total (months) | 12.0 ± 6.6 | 10.8 ± 6.5 |
| Never-PA | 12.2 ± 6.6 | 11.0 ± 6.6 |
| Initiation-PA | 12.0 ± 6.6 | 10.9 ± 6.5 |
| Withdrawal-PA | 12.2 ± 6.5 | 10.8 ± 6.5 |
| Maintenance-PA | 11.8 ± 6.6 | 10.5 ± 6.5 |

Pre-MCI diagnosis period defined as the period from 1^st^ health examination to MCI diagnosis

Post-MCI diagnosis period defined as the period from MCI diagnosis to 2^nd^ health examination

PA=physical activity

Supplementary table 2. Baseline characteristics of the study population between Irregular and Regular-PA

|  | Irregular-PA (n=197619) | Regular-PA (n=49530) | p value |
| --- | --- | --- | --- |
| Age, year | 67.3 ± 9.4 | 65.9 ± 8.7 | <0.0001 |
| Female, n (%) | 60616 (30.7) | 20500 (41.4) | <0.0001 |
| Hypertension, n (%) | 110215 (55.8) | 26591 (53.7) | <0.0001 |
| Diabetes mellitus, n (%) | 42583 (21.6) | 10640 (21.5) | 0.749 |
| Dyslipidemia, n (%) | 90539 (45.8) | 22907 (46.3) | 0.0832 |
| Obesity*, n (%) | 72604 (36.7) | 17676 (35.7) | <0.0001 |
| Body mass index, kg/m^2^ | 24.15 ± 3.16 | 24.14 ± 2.89 | 0.3901 |
| Smoking, n (%) |  |  | <0.0001 |
| Non-smoker | 158332 (80.1) | 36677 (74.1) |  |
| Ex-smoker | 23980 (12.1) | 9279 (18.7) |  |
| Current smoker | 15307 (7.8) | 3574 (7.2) |  |
| Drinking, n (%) |  |  | <0.0001 |
| Non-drinker | 160266 (81.1) | 36543 (73.8) |  |
| mild to moderate drinker | 32096 (16.2) | 11289 (22.8) |  |
| heavy drinker | 5257 (2.7) | 1698 (3.4) |  |
| Time to conversion from MCI to dementia (months) | 31.3 ± 16.7 | 31.3 ± 16.6 | 0.8199 |

PA=physical activity

* body mass index ≥ 25 kg/m2

Supplementary table3. Interactions of comorbidities with continuity of physical activity

| Comorbidity |  | Group | HR(95% C.I) | p for interaction |
| --- | --- | --- | --- | --- |
| Hypertension | No | Never-PA | 1(Ref.) | 0.811 |
|  |  | Initiation-PA | 0.875(0.825,0.929) |  |
|  |  | Withdrawal-PA | 0.968(0.915,1.025) |  |
|  |  | Maintenance-PA | 0.801(0.754,0.851) |  |
|  | Yes | Never-PA | 1(Ref.) |  |
|  |  | Initiation-PA | 0.897(0.855,0.94) |  |
|  |  | Withdrawal-PA | 0.986(0.943,1.03) |  |
|  |  | Maintenance-PA | 0.795(0.756,0.836) |  |
| Diabetes mellitus | No | Never-PA | 1(Ref.) | 0.3039 |
|  |  | Initiation-PA | 0.892(0.855,0.932) |  |
|  |  | Withdrawal-PA | 0.999(0.959,1.04) |  |
|  |  | Maintenance-PA | 0.799(0.763,0.836) |  |
|  | Yes | Never-PA | 1(Ref.) |  |
|  |  | Initiation-PA | 0.879(0.817,0.945) |  |
|  |  | Withdrawal-PA | 0.929(0.868,0.995) |  |
|  |  | Maintenance-PA | 0.797(0.738,0.86) |  |
| Dyslipidemia | No | Never-PA | 1(Ref.) | 0.4697 |
|  |  | Initiation-PA | 0.907(0.862,0.953) |  |
|  |  | Withdrawal-PA | 0.998(0.952,1.046) |  |
|  |  | Maintenance-PA | 0.81(0.768,0.854) |  |
|  | Yes | Never-PA | 1(Ref.) |  |
|  |  | Initiation-PA | 0.867(0.821,0.917) |  |
|  |  | Withdrawal-PA | 0.957(0.908,1.008) |  |
|  |  | Maintenance-PA | 0.783(0.739,0.83) |  |
| Obesity* | No | Never-PA | 1(Ref.) | 0.7232 |
|  |  | Initiation-PA | 0.876(0.838,0.916) |  |
|  |  | Withdrawal-PA | 0.979(0.939,1.021) |  |
|  |  | Maintenance-PA | 0.797(0.761,0.835) |  |
|  | Yes | Never-PA | 1(Ref.) |  |
|  |  | Initiation-PA | 0.913(0.854,0.976) |  |
|  |  | Withdrawal-PA | 0.974(0.914,1.039) |  |
|  |  | Maintenance-PA | 0.79(0.736,0.849) |  |

* body mass index ≥ 25 kg/m2
